# The Contribution of Microorganisms to the Quality and Flavor Formation of Chinese Traditional Fermented Meat and Fish Products

**DOI:** 10.3390/foods13040608

**Published:** 2024-02-17

**Authors:** Jingjing Mao, Xinyi Wang, Hongfan Chen, Zhiping Zhao, Dayu Liu, Yin Zhang, Xin Nie

**Affiliations:** 1 Meat Processing Key Laboratory of Sichuan Province, College of Food and Biological Engineering, Chengdu University, Chengdu 610106, China; 2 College of Food Science and Technology, Sichuan Tourism University, Chengdu 610100, China

**Keywords:** Chinese traditional meat products, spontaneous fermentation, flavor, quality, metabolic pathways

## Abstract

Guizhou sour meat and sour fish, Chaoshan fish sauce, Sichuan sausage and bacon, Cantonese sausage, Jinhua ham, and Xinjiang air-dried beef are eight representatives of Chinese traditional fermented meat and fish products (FMFPs), which are favored by Chinese consumers due to their high nutritional value and quality. The quality of the spontaneously fermented Chinese traditional FMFP is closely correlated with microorganisms. Moreover, the dominant microorganisms are significantly different due to regional differences. The effects of microorganisms on the texture, color, flavor, nutrition, functional properties, and safety of Chinese traditional FMFPs have not been not fully described. Additionally, metabolic pathways for flavor formation of Chinese traditional FMFPs have not well been summarized. This article describes the seven characteristic Chinese traditional FMFPs and correlated dominant microorganisms in different regions of China. The effects of microorganisms on the texture, color, and flavor of Chinese traditional FMFPs are discussed. Furthermore, the metabolic pathways of microbial regulation of flavor formation in Chinese traditional FMFPs are proposed. This work provides a theoretical basis for improvement of Chinese traditional FMFPs by inoculating functional microorganisms isolated from Chinese traditional fermented foods.

## 1. Introduction

Fermented meat products and fish products (FMFPs) are a kind of meat product with special flavor, color, texture, nutritional value, and shelf life produced by spontaneous or inoculated fermentation [1]. Global meat consumption has exceeded 300 million tons per year, among which more than 30% are meat products, with FMFPs being the most important kind of meat products [2]. In contrast to foreign fermented meat products, which are mostly fermented with a starter, most fermented meats in China nowadays are naturally fermented and various spices are added during the fermentation process, so there is a significant difference between the flavor, taste, and texture of the products and those of foreign fermented meats. Chinese FMFPs have strong local and geographical characteristics, such as Guizhou sour meat, Guizhou sour fish, Chaoshan fish sauce, Sichuan sausage, Sichuan bacon, Jinhua ham, and Xinjiang air-dried beef. Due to the differences in raw materials, processing, and fermentation environments, the microbiota of FMFPs in different regions are complex [3]. For example, the main bacteria of smoked sausage and air-dried sausage in China are *Staphylococcus* spp. and *lactic acid bacteria* (LAB) [4]. *Aspergillus*, *Saccharomyces*, *Staphylococcus*, and *Corbettella* are the dominant microorganisms in post-cooked Jinhua ham [5].

During processing, a certain number of microorganisms can effectively increase the content of bioactive ingredients in meat [6]. FMFPs have a distinctive product quality owing to the abundant activity of microorganisms, as well as a high number of free amino acids (FAAs) and peptides produced during fermentation [7]. Moreover, microorganisms can induce dramatic changes in the color, flavor, and nutrition of FMFPs. Due to the wide variety of microorganisms produced in natural fermentation in Chinese FMFPs, therefore, it is necessary to understand how microorganisms affect the quality of Chinese FMFPs. The effects of microorganisms on the quality, such as texture, color, flavor, nutritional value, functional properties, and safety of Chinese FMFP, are not well summarized. Additionally, metabolic pathways for flavor formation of FMFP have not been reviewed. This work describes the dominant microorganisms and their effects on the quality of Chinese FMFPs. In addition, the regulation of metabolic pathways for flavor formation of FMFPs has also been investigated.

## 2. Chinese Local Traditional FMFPs and Correlated Dominant Microorganisms

With China’s wide geographical area, different geographical and environmental conditions have developed different types of fermented meat products with different flavors, which are characterized by attractive colors, unique flavors, and rich nutrients. Therefore, this paper introduces eight types of traditional FMFP from south to north of China with Chinese characteristics. The dominant genera of different fermented meat and fish products are presented in Table 1.

### 2.1. Guizhou Sour Meat

Guizhou sour meat is a special FMFP of the Chinese Dong, Miao, and Buyi Minorities with a unique flavor, a high concentration of active peptides and nutrients, and a long preservation time. To process the sour meat, the pre-processed raw meat (pork) is sliced into thin slices (0.3–0.5 cm), salt and chili powder are added, and it is then sealed into a suitable container (fir barrel or jar) followed by spontaneous fermentation for 20–30 days. During spontaneous fermentation, a large number of microbial colonies of different genera grow and thus lead to diverse metabolic characteristics depending on different production environments and processes. The common dominant bacteria in the traditional sour meat include *Lactobacillus*, *Floccus*, *Staphylococcus*, and *Micrococcus* (*M.*). In addition, fungal microorganisms such as *Hansenula* spp. and *Picococcus* spp. are also the core microorganisms in sour meat [8]. During fermentation, the sour meat produces a large number of flavor substances, such as esters, alcohols, acids, FAAs, and antioxidant peptides, owing to the functions of microorganisms and endogenous enzymes. LAB will dominate the fermentation system, which can enhance the quality of sour meat by creating a condition that is favorable for LAB growth [9]. However, spontaneous fermentation usually causes unstable quality of sour meat. Consequently, starter cultures have been attracted more and more attention from researchers. Jiang et al. screened two strains with strong lipase production activity, *Staphylococcus* (*S.*) *epidermidis* N30 and *Yarrowia lipolytica* C11, which were found to be effective in enhancing the flavor content of sour meat [10]. On the other hand, *Lactobacillus* (*L.*) *curvatus* LAB26 and *L. glutamicus* SWU73571 isolated from sour meat could effectively increase the FAA content and inhibit the production of harmful substances such as nitrite and biogenic amine [11]. Furthermore, it has also been demonstrated that inoculation with *Saccharomyces cerevisiae* LSXPSC1 can improve the sensory properties and increase the concentration of FAAs and volatile flavors in sour meat [12]. The sensory qualities of fermented meat could also be enhanced by *L. plantarum* CMRC6 and *Staphylococcus* spp. SX16 isolated from sour meat with excellent proteolytic activities [13].

### 2.2. Guizhou Sour Fish

Besides sour meat, sour fish is another most characteristic Guizhou FMFP with strong local ethnic minority characteristics, and is very popular in the Miao, Dong, Tujia, and other ethnic groups. Sour fish is usually made from carp or grass carp. After slaughter and pickling, the fish meat is mixed with fried grains (glutinous rice or cornmeal) and then hermetically sealed and fermented at room temperature for approximately 35 days [14]. Customers like the flavorful and refreshing fermented sour fish for its rich flavor and nutrients [15]. The quality of the traditional fermented fish mainly depends on the microorganisms originating from raw materials and environments. In general, the distinctive aroma of fermented fish products is mainly derived from the degradation of proteins, fats, and carbohydrates into esters, alcohols, acids, aldehydes, ketones, and other aromatic compounds. During sour fish fermentation, microbial interactions play an important role in flavor metabolism. For example, *Lactobacillus* spp. has a higher abundance throughout the fermentation, which not only promotes flavor formation but also inhibits the growth of spoilage microorganisms [16].

The microorganisms affecting the characteristic flavors of sour fish in the pre-fermentation and post-fermentation periods are different. *Lactobacillus* spp., *Staphylococcus* spp., and *Megacoccus* spp. are predominant in the pre-fermentation period, while the relative abundance of *Wickerhamomyces anomalus* and *Candida* spp. gradually increases in the post-fermentation period. It has been shown that microbial community succession and microbial metabolisms are closely related to the quality of sour fish products [17]. The *L. plantarum* 120, *L. plantarum* 145, *Pediococcus* (*P.*) *pentosaceus* 220, and *S. xylosus* 135 isolated from sour fish could significantly increase the essential amino acids and decrease the contaminating microorganisms of sour fish [18].

### 2.3. Chaoshan Fish Sauce

Fish sauce, also known as nuoc-mam, originated from Fuzhou located in Fujian Province and Chaoshan located in Guangdong Province, and is an aquatic condiment made from low-value fish and shrimp or by-products of aquatic products. Fresh fish is usually gutted and cut into pieces, sufficient salt is sprinkled evenly over the surface, it is mixed well, and seasonings such as garlic, chili peppers, ginger are added to enrich the flavor. Then, the treated fish is placed in bottles with an adequate amount of water followed by anaerobic fermentation for a couple of months [19]. Fish sauce is abundant in essential amino acids, minerals, vitamins, and other nutrients, with the functions of lowering blood pressure and cholesterol and providing antioxidants [20]. The formation of traditional fish sauce flavor is a dynamic and complex biochemical process determined by the microbial composition and metabolic activity under a variety of fermentation conditions [21]. It has been confirmed that 30 microorganisms are closely associated with the volatile flavor substances of fish sauce, among which *Halomonas*, *Tetragenococcus*, and *Trichococcus* are closely associated with various volatile flavor substances. The primary contributors to the fermentation process of fish sauce and also the key microorganisms involved in the production of flavor compounds are *Halanaerobium*, *Halomonas*, *Tetragenococcus*, *Halococcus*, and *Candidatus Frackibacter* [22]. Inoculation of *Halanaerobium* (*H.*) *fermentans* YL9-2 could significantly increase the amino acid nitrogen of fish sauce, reduce the formation of biogenic amines, and thus effectively improve the quality and flavor of fish sauce [23].

### 2.4. Sichuan Sausage

As a typical representative of FMFPs, sausages vary from area to area depending on their geographical locations and lifestyles in China. Among them, Sichuan sausage is widely accepted for its spicy and unique flavor. Distinguished from other sausages, Sichuan sausage is usually made from pork with a fat-to-lean ratio of 3:7, which is combined with seasonings such as salt, baijiu, pepper powder, chili powder. The sausage is usually divided into sections of about 15 cm in length by tying a knot with a string, and is then air dried for about 7–10 days. These traditional fermented sausages are unique, nutritious, and very popular among the general population [4]. The flavor of Chinese traditional sausages is produced by the physicochemical reactions and microbial metabolisms during the fermentation and maturation process. Their unique flavor is related to microorganisms such as LAB, staphylococci, and yeast. The microbial diversity and succession regulations during spontaneous fermentation play very important roles in the flavor development of Sichuan sausage. Bacteria are considered the dominant microbial community in the fermentation process, while fungi are relatively rare [24]. The primary reason for the involvement of LAB in the fermentation process of sausages is their impact on carbohydrate metabolism, resulting in a significant reduction in pH [25]. *L. plantarum* and *L. casei* are the starters that are mostly used for fermented sausage, and can effectively degrade myogenic fibrin and sarcoplasmic fibrin [26]. Inoculation of *L. plantarum* XC-3 could reduce the pH and residual nitrite of fermented sausages while increasing the total volatile flavor substances [27]. The bacteria in Chinese sausage mainly consists of *Lactobacillus*, *Staphylococcus*, and *Weisseria* as the dominant genera, which closely correlate with the production of flavor substances [28].

### 2.5. Cantonese Sausage

Similarly to Sichuan sausage, Cantonese sausage is also popular among many consumers because of its sweet taste [29]. As a traditional meat product specializing in the Guangdong region of China, the biggest difference between Cantonese sausage and Sichuan sausage lies in the addition of supplementary ingredients, which is required to make Cantonese sausage, to which white sugar and Baijiu are added. Typically, the processing involves cutting the pork into 2 cm strips according to the pork fat-to-lean ratio of 2:8 or 3:7, then adding 10% sugar, 2% white wine, and mixing well to marinate for about 15 min, until all of the sugar and salt is melted and absorbed. The mixture is filled into natural pig casings and naturally air dried for 4–7 days [30]. The flavor of Cantonese sausage mainly comes from the flavor of the auxiliary ingredients themselves, the degradation and oxidation of proteins and lipids, and the biochemical reactions caused by the extracellular enzymes secreted by microorganisms. The microbial metabolism of Cantonese sausage also plays a key role in flavor formation during processing, storage, etc. [31]. Currently, the production process of Cantonese sausage does not include the addition of extra starter cultures. However, a large number of microorganisms are involved in the process of processing and storage, which generate a variety of flavor substances during fermentation. Among them, staphylococci and micrococci are the main dominant microbial groups in the fermentation process of Cantonese sausage [32]. Traditional Cantonese sausages are highly susceptible to the growth of harmful microorganisms due to their own flora dominance, making it difficult to ensure product quality and safety. Artificially inoculated microbial starter cultures can be used to induce the fermentation process, inhibit the growth of pathogenic microorganisms and the synthesis of potentially harmful compounds (e.g., biogenic amines), and ensure the safety of the final product [33]. Wu et al. isolated *S. condimenti* from Cantonese sausage and inoculated it into Cantonese sausage, and found that this strain facilitated the degradation and oxidation of fats and proteins, leading to the production of more flavor substances and the improvement in the flavor of Cantonese sausage [34]. In addition, Wang et al. demonstrated that the addition of mixed starter cultures (*L. sakei*, *P. pentosaceus*, *S. xylosus*, and *S. carnosus*) was beneficial for improving the microbiological quality and food safety of Chinese sausages [35].

### 2.6. Sichuan Bacon

Traditional Sichuan bacon is a kind of FMFP, and is favored by Chinese people for its unique flavor, distinctive color, and rich aroma [36]. To process Sichuan bacon, fresh pork is cut into 30 cm lengths and 5 cm widths, and then mixed well with seasonings (salt, cooking wine, sugar, and pepper). The cured strips are smoked with conifer wood to color the bacon, and finally air dried outside. During processing and storage, microorganisms promote color and flavor formation by metabolizing protein, fat, and other nutrients [37]. Due to the relatively open environment, a variety of microorganisms are involved in the ripening process, which greatly influences the quality of the traditional bacon. The microbial diversity of Sichuan bacon is significantly different from that of other regions, and *Staphylococcus* spp. is clearly the dominant genus in Sichuan bacon [38]. During processing, the surfaces of raw meat are exposed to the environment; therefore, the environmental microorganisms participate in the ripening process, which probably leads to spoilage. *S. xylosus*, *Carnobacterium maltaromaticum*, *Leuconostoc mesenteroides*, *Serratia liquefaciens*, and *Leuconostoc gelidum* are the predominant microorganisms responsible for bacon spoilage [39]. Differential metabolites in bacon are strongly correlated with dominant bacterial species, such as *Salinivibrio*, *Vibrio*, *Cobetia*, and *Staphylococcus* [40]. Due to the specific microbial ecosystem in bacon, a rich and diverse fungal population is currently found in bacon, including *Aspergillus*, *Mucor*, *Penicillium*, and yeast. Some of these fungi can lead to favorable flavor, antioxidant effects, and protection against harmful microorganisms, while some others can result in undesirable effects such as unpleasant colored spots, off-flavors, or toxic fungal metabolites. For example, *Aspergillus*, *Penicillium*, and *Xylella* in bacon are positively correlated with fat content and pH in the production of lipase [41].

### 2.7. Jinhua Ham

Jinhua ham is one of the representative products of Chinese dry-cured ham made from pigs’ hind legs [42], and has been widely acclaimed as one of the top three traditional hams in China. Jinhua ham is made from the famous Chinese pig “Jinhua Liangtou wu”, which have a black head and tail. The special climatic conditions of Jinhua region and the curing process inherited from generation to generation for thousands of years make it unique. Jinhua ham is usually made from fresh pork hind legs, which are trimmed and flattened. The surface of the hind legs is coated with salt, once every 2–3 days, and repeated 6–7 times. The hams are then soaked in 10 °C water for 10 h, and brushed to remove salt and stains from the surface, and then soaked in water for 2 h. Finally, the ham is air dried in the sun for 5–6 days, until hardened and hung in a ventilated dry place; fermentation usually takes half a year [43]. In addition to Jinhua ham, Xuanwei ham and Nuodeng ham produced in Yunnan Province are also very popular because of the geographical advantages of low temperatures and sufficient sun light. During fermentation, microorganisms promote the production of the aroma in ham and effectively prevent its spoilage, thus prolonging the product’s shelf life. Spoilage microorganisms in ham have been extensively investigated. It has been shown that *Enterobacteriaceae farmei* CDC 2991-81, *Bacillaceae cereus* ATCC 14579, and *Enterococcaceae faecalis* ATCC 19433 are the main microorganisms that cause the spoilage of Jinhua ham [44]. In addition, the dynamic changes in microbial communities during the whole fermentation of ham also have to be investigated.

The dominant microorganisms in Jinhua ham are *Staphylococcus* spp. and *Tetrahymena* spp., which are conducive to producing flavor substances such as nonanal and benzaldehyde [45]. For example, isolated *Staphylococcus* with high protein hydrolytic and lipolytic activity is associated with aldehyde content, demonstrating the contribution of staphylococci to the flavor formation process of Jinhua ham [46]. Regarding its unique flavor, microorganisms with specific functions can be selectively isolated from ham and inoculated in ham, and can effectively improve the product quality. *L. fermentum* YZU-06 isolated from Jinhua ham can be used as a starter to improve ham flavor [47]. In addition, not only does the ham have a unique flavor, but the fatty acids in Jinhua ham can prevent alcohol-associated liver damage by regulating the gut microbiota and improving the intestinal barrier [48].

### 2.8. Xinjiang Air-Dried Beef

Air-dried beef is a traditional food in the Kazakh region of Ili Prefecture, Xinjiang. When processing Xinjiang air-dried beef, the beef is cut into strips of about 50 cm in length and 15 cm in width and then coated with salt. The meat is then air dried at a temperature of 0–5 °C for 15 days. The formation of its flavor quality is similar to that of Chinese traditional sausage. Microorganisms from the raw materials and the surrounding environments, particularly some cold-resistant LAB and staphylococci, continuously participate in vigorous metabolic activities during air drying. These activities catalyze the production of acid and bacteriocin production, inhibit bacteria, and produce lipases and proteases to degrade proteins and oxidize lipids, thereby improving the taste and edible quality of the finished product [49]. The fermentation process of air-dried beef is characterized by complex microbial community succession, in which the enzymes produced by fungus decompose sugars, fats, and proteins to flavor substances such as ketones, aldehydes, and esters [50]. Air-dried beef is distinguished from other traditional FMFPs by the absence of spices. Therefore, there are less volatile flavor substances compared to other FMFPs with abundant spices. However, microorganisms also play important roles in the quality and safety of the air-dried beef. *Bacillus* spp. and *Cyclospora* spp. are proposed to be the dominant microorganisms during beef air drying [51]. The raw materials of air-dried meat are mostly not sterilized, and the surrounding environmental conditions are unstable, which are the mainly factors causing the air-dried beef to be contaminated by pathogenic or spoilage bacteria. Identification of spoilage microorganisms in air-dried beef has not been reported [52]. Therefore, intensive studies are required to be performed to understand the dynamics of microbiota and spoilage processes during fermentation to improve the product.

**Table 1:** The dominant genera of Chinese characteristic FMFPs.

Fermented Meat and Fish Products	Predominant Genera	References
Guizhou sour meat	*Lactobacillus*, *Weiss*, *Lactococcus*	[53]
Guizhou sour fish	*Lactobacillus*, *Megacoccus*, *Staphylococcus*	[53]
Chaoshan fish sauce	*Tetragenococcus*, *Carnobacterium*, *Lentibacillus*	[54]
Sichuan sausage	*Lactobacillus*, *Weissella*, *Pediococcus*	[24]
Cantonese sausage	*Staphylococci*, *micrococci*	[32]
Sichuan bacon	*Aspergillus*, *Debaryomyces*, *CandidaStaphylococcus*, *Macrococcus*, *Acinetobacter*	[55]
Jinhua ham	*Aspergillus*, *Saccharomyces*, *Staphylococcus*, *Cobetia*	[5]
Xinjiang air-dried beef	*Lycobacterium*, *Cyclosphaera*	[51]

## 3. Effects of Microorganisms on the Quality of Chinese Traditional FMFPs

In China, different regional characteristics and human customs profoundly affect the processing of FMFPs. Normally, FMFPs are marinated with a large amount of salt and spices, and microbial starter cultures can better promote the improvement of flavor than other food additives, such as sodium pyrophosphate, modified starch, and soy protein. The flavor of Chinese traditional FMFPs mostly comes from the maturation of flavor in the post fermentation period. Microbial growth and reproduction in the post-fermentation period, and abundant microbial activities, will directly affect the metabolism of protein, lipolysis, and carbohydrates in the product, which in turn affects the quality of the product. Therefore, the effect of microorganisms on the quality of Chinese traditional FMFPs needs to be further explored.

### 3.1. Effect of Microorganisms on the Texture of Chinese Traditional FMFP

Texture, as a visual indicator of the sensory quality of a meat product, is influenced by microorganisms and enzymes [56]. Gelatinization and denaturation of muscle proteins promote the textural alterations that frequently occur in Chinese characteristic FMFPs. Because of natural fermentation, Chinese characteristic FMFPs usually produce abundant microorganisms, resulting in a weakly acidic environment that induces gelatinization of meat products [57]. Consequently, addition of starter cultures is a promising method for enhancing meat tenderness and overall qualities. The composition of beneficial microorganisms in various fermented products will change based on any particular methods of fermentation procedures and the environmental conditions. To enhance product quality, it is possible to inoculate diverse functional microorganisms into meat products. This approach not only preserves the distinctive flavor inherent to the FMFP, but also introduces additional beneficial microorganisms bringing favorable improvements to the products. For example, the *P. parvulus* isolated from Xuanwei ham could significantly improve the ham texture [58]. The *L*. *pentosus* 31-1 isolated from Xuanwei ham could not only cause a concentration of substantial decrease in the presence of *Listeria* (*L.*) *monocytogenes* and *S. aureus*, but also yield a more visually appealing surface, improved texture, and enhanced sensory attributes [59]. The *S. xylosus* P2 isolated from Chinese bacon made beef jerky more attractive in terms of color and texture [60]. According to Zhao et al., the microstructure of Chinese characteristic fermented spicy rabbit showed considerable variations when *L. paracasei* was added, leading to a substantial improvement in tenderness [61].

### 3.2. Effect of Microorganisms on the Color of Chinese Traditional FMFPs 

The color of Chinese characteristic FMFPs mostly tends to be reddish brown due to natural air drying. Therefore, brightening the color of Chinese characteristic meat products is an important measurement for quality improvement. In the processing and storage of meat and meat products, the proliferation of microorganisms is a major factor affecting the color. Some of these microorganisms, such as lactobacilli, staphylococci, and yeast, can convert NO_3_^−^ into NO, which combines with myoglobin in meat to produce nitrosomyoglobin, maintaining the color of the product [62]. Yeasts can influence the color and aroma of dry fermented sausages through oxygen-scavenging and lipolytic activities [63]. Ras et al. found a *nos* gene encoding nitric oxide synthase (NOS) in the genome of all staphylococci, especially coagulase-negative staphylococci, where NO binds to the heme iron atom of myoglobin to form nitrated myoglobin, the source of the red pigment in FMFPs [64]. By inoculating coagulase-negative staphylococci in dried sausages, Huang et al. found that this strain had NOS activity, which could catalyze the hydroxylation of L-arginine to produce L-citrulline and NO. Then, NO could bind to myoglobin to form bright red-colored nitrosomyoglobin, thus significantly increasing the redness of dried sausages. The color of the fermented sausage will be brighter and redder with the addition of *S. lignus* LQ3 compared to the control without the starter [65]. In addition, spoilage of fermented meat, leading to product discoloration, is also caused by microorganisms, some of which metabolize proteins into other compounds such as dimethylamine and trimethylamine, resulting in color changes.

### 3.3. Effect of Microorganisms on the Flavor of Chinese Traditional FMFPs

One criterion for judging FMFPs is flavor, which is impacted by starter cultures, the processing methods, and the ingredients. Additionally, a variety of flavor substances, including lactic acid, FAAs, and aromatic compounds, determine the product’s final flavor [66]. The distinctive flavor of Chinese meat products is a result of the gradual enzymatic oxidation of proteins and lipids during the natural air drying and microbial fermentation. Among the processes, the decomposition and oxidation of fat are the essential ways to develop the flavor [67]. Additionally, microorganisms have a significant influence on the formation of volatiles in fermented meats. *Lactobacillus*, the dominant microorganism in most fermented meats, is also often used as a starter culture for sausage and bacon production. The function of LAB is to decompose carbohydrates and metabolize them into lactic acid, which rapidly acidifies the food matrix. Most of the LAB can promote the formation of the fermented food flavor [68]. It has been demonstrated that *Weissella hellenica* HRB6 and *L. sakei* HRB10 correlated with most of the key volatile compounds in sausage [69]. In addition, *P. pentosaceus* isolated from Harbin dry sausage could be an excellent starter culture for the production of a rich flavor using myostatin protein [70]. *Staphylococcus* mainly promotes the production and stabilization of myoglobin, prevents harmful bacteria, and inhibits oxidative rancidity, particularly promoting the characteristic flavor of FMFPs under the catalysis of nitrate reductase. The capacity of staphylococci to enzymatically degrade proteins and lipids shows species-specific variations, and its significance in the development of flavor is of utmost importance. Moreover, *Staphylococcus* can generate aroma substances to improve flavor. The *S. condimenti* and *M. caseolyticus* isolated from Chinese Cantonese sausage can accelerate fat and protein oxidation and thus improve the flavor profile of the sausage [34]. The *S. xylosus* YCC3 can promote the development of (Z)-hept-2-enal, (E)-2-octenal, 1-nonenal, and octanal volatile flavor compounds, and 1-octen-3-ol was found in the inoculated sausages compared to the control [71]. In fermented sausage, the species of mold will vary according to the processing and environment. Mold contributes to hydrolyze starch and protein through the endogenous amylase and protein hydrolase to accelerate the fermentation process [72]. *Aspergillus oryzae* is an important source of flavor in the pre-fermentation of fish sauce and is also the main strain used in traditional Chinese soy sauce brewing [73]. *Penicillium* is often used as a starter in fermented sausages, e.g., *P. salami* ITEM 15302 is a fast-growing mold on dry cured sausage casings, and has high lipolytic and proteolytic enzyme activity, which gives typical organoleptic characteristics to meat products [74]. However, some molds can also contaminate products with ochratoxin A, such as *P. nordicum* and *P. verrucosum* [75].

### 3.4. Effect of Microorganisms on the Nutrition of Chinese Traditional FMFPs

Chinese characteristic meat products are considered a traditional health food compared with ordinary meat products, and are more nutritious and rich in a variety of vitamins, minerals, and essential amino acids [76]. During the maturation of fermented meat, specific microbiota are produced, and probiotic metabolites are consequently formed [77]. Amino acids in fermented meat, such as taurine (a sulfur-containing β-amino acid), creatine (a metabolite of arginine, glycine, and methionine), carnosine (a dipeptide; β-alanyl -L-histidine), and 4-hydroxyproline (also often referred to as an amino acid), may improve neurological abnormalities and promote human health [78]. Meat products are enriched with active prebiotics and probiotics, which can improve the nutritional and health value of FMFPs [79]. *P. pentosaceus*, *S. xylosus*, and the combination of with *P. pentosaceus* and *S. xylosus* could significantly improve the FAAs and saturated fatty acid of sausage [80]. Amino acid and other nutrients in fermented meat are produced from protein degradation. Microbial activity will promote the release of amino acids, and aromatic amino acids can be metabolized into flavor substances by transaminases. However, if highly oxidized, it will affect the digestive utilization of fermented meat, and then the nutritional value of fermented meat will be reduced [25]. Cao and co-workers found that the inoculation of *L. plantarum* CD101 and *S. simulans* NJ201 resulted in a significant reduction in protein oxidation throughout the fermentation process and thus altered the product nutrition [81].

### 3.5. Effect of Microorganisms on the Functional Properties of Chinese Traditional FMFPs

China has long been skilled in the application of traditional fermentation techniques in the production of meat products. As a result, Chinese characteristic meat products not only perform excellently in terms of flavor but also contain a variety of functional properties that are beneficial to the human body. Characterization of functional microorganisms in FMFPs contributes to a deeper understanding of the efficacy of FMFPs. *Lactobacillus* exhibits antioxidant properties in the host intestine and promotes the production of antioxidant enzymes, therefore removing reactive oxygen species (ROS). The regulation of LAB antioxidant properties is complicated, and includes regulation of the oxidation-reduction system, production of antioxidant metabolites, scavenging of free radicals, and chelating of metal ions [82]. The antioxidant activity of the sausages inoculated with *L. plantarum* CD101 significantly increased compared to the control [81]. *Lactobacillus*, a probiotic possessing a variety of beneficial functions, probably plays a role in lowering cholesterol in FMFPs. Cholesterol is a derivative of cyclopentane polyhydrophenanthrene. One potential mechanism through which *Lactobacillus* may reduce cholesterol levels is by producing bile salt hydrolase (BSH), which has the ability to separate bile salts in the hepatic–intestinal cycle and can decompose bile salts bound to taurine or glycine into free bile salts and amino acid residues. The synthesis of bile acids requires cholesterol as a precursor substance, and this process ultimately leads to a decrease in the serum cholesterol concentration. Ding et al. screened 18 strains of cholesterol-lowering LAB from fermented sour meat, among which strain SR10 had the strongest cholesterol-lowering ability, with a cholesterol reduction rate of 33.78% [83]. In addition, *Lactobacillus* spp. and *Bifidobacterium* spp., both of which exhibit significant cholesterol-lowering activity in the body, are effective in lowering total serum cholesterol by producing bile salt hydrolase and accelerating the decomposition of cholesterol [84]. It has been shown that probiotics such as *Lactobacillus* also have significant efficacy in reducing hyperglycemia, hyperlipidemia, and hypertension, and have non-toxic side effects compared with traditional drugs [85]. In addition, dry-cured meat products contain antioxidant peptides that convert free radicals into more stable products and terminate the free radical chain reaction, thereby improving product quality and durability [86].

### 3.6. Effect of Microorganisms on the Safety of Chinese Traditional FMFPs

Dry-cured meat products account for a large proportion of Chinese characteristic FMFPs. The process of curing may lead to food safety problems, such as excessive biogenic amines and nitrites, due to the extensive metabolism of microorganisms and the environmental problems. Biogenic amines are generated from FAAs through removal of the α-carboxyl group by amino acid decarboxylase, leading to diarrhea, headache, and other uncomfortable symptoms when they are in excess in the body [87]. The main types of biogenic amines found in meat products are spermidine, tyramine, histamine, etc. [88,89]. Of these, the heterocyclic biogenic amine histamine is the most toxic, and can lead to neurotoxicity [90]. It has been commonly believed that irradiation treatment is an effective way to control biogenic amines, but it is not widely applied owing to its high cost and effects on the flavor of the product. Therefore, microbial degradation techniques are being frequently applied in production. Selecting starter cultures with amine oxidase-producing activity and without decarboxylase activity is an important solution to reduce its accumulation. It is well known that *Lactobacillus*, *Lactococcus*, and *Micrococcus* have amine oxidase-producing activity. *Weissella viridescens* F2 and *Lactiplantibacillus plantarum* His6 benefit the direct degradation of biogenic amines and inhibition of the growth of amine-producing bacteria [91]. *S. pasteuri* Sp, *S. epidermidis* Se, *S. carnosus* Sc1, *S. carnosus* Sc2, and *S. simulans* Ss exhibit a significant reduction in biogenic amines and have the potential to be utilized as starter cultures to control biogenic amines in FMFPs [92].

In addition to biogenic amines, spoilage bacteria are also found in FMFPs, and spoilage of meat products is caused by the growth activities of their dominant spoilage bacteria. The common pathogenic microorganisms in fermented meat are *L. monocytogenes*, *Salmonella*, diarrheagenic *Escherichia coli*, etc. Francesca et al. inoculated Chinese sausage with *L. sakei* and *S. xylosus* and found that these bacteria were effective in inhibiting the growth of food-borne bacteria such as *L. monocytogenes* and Gram-negative spoilage bacteria [93]. *Lactobacillus* can inhibit the growth of disease-causing or spoilage microorganisms by rapidly producing acid to increase the acidity of fermented meat and generating bacteriocins [94].

Nitrite residue, as an important factor in measuring the safety of fermented meat, can produce toxic nitrosamines when in overabundance, and can be effectively reduced by the addition of microorganisms [95]. It has been found that *L. plantarum* isolated from Chinese fermented sausages could not only improve the color of sausages but also effectively reduce the nitrite content of the products [96]. In addition, the nitrate reductase activity of microorganisms can be utilized, which can be an alternative to artificial addition of nitrite and effectively improve the safety of meat products [97].

## 4. Metabolic Pathways of Microbial Regulation of Flavor Formation in Chinese FMFPs

**Figure 1:**
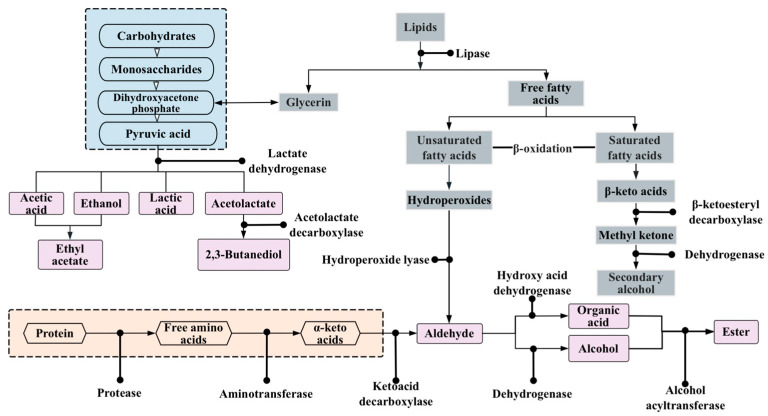
Metabolic pathways of the microorganisms affecting the formation of flavors of carbohydrates, proteins, and lipids.

Flavor is considered to be one of the most important characteristics determining the acceptance of food by consumers. The major volatile flavor substances in FMFPs include alcohols, aldehydes, esters, ketones, acids, alkylenes, aromatic hydrocarbons, sulfur-containing compounds, nitrogen-containing compounds, and terpenes [98]. Carbohydrates, lipids, proteins, and other macronutrients are the important sources of flavor substances. These substances are hydrolyzed under the synergistic action of microorganisms and endogenous enzymes to produce primary metabolites such as monosaccharides, free fatty acids (FFAs), and FAAs, and further metabolized to produce various secondary aromatic compounds. Microorganisms are involved with the metabolic pathways involved in the production of flavor substances during fermentation (Figure 1).

### 4.1. Proteolytic Metabolic Pathways

The protein hydrolysis pathway is largely dependent on endogenous and microbial enzymes. These enzymes hydrolyze sarcoplasmic and myogenic stringy proteins and induce FAAs through hydrolysis. The process serves as a precursor material for the production of aromatic compounds in FMFPs [99]. In FMFPs, proteins are decomposed into amino acids and subsequently metabolized through a series of pathways to produce volatile flavor substances [100]. The final products of protein digestion are primarily amino acids. Additionally, small amounts of tiny peptides are included, all of which are absorbed by small intestinal mucosa. However, the small peptides are hydrolyzed into FAAs by peptidases in the cytoplasm. Amino acids have been subjected to deamidation to produce α-keto acids, which are either synthesized as non-essential amino acids, converted to sugars or fats, or converted to intermediate products of the tricarboxylic acid cycle and oxidized for energy supply. *Debaryomyces* (*D.*) *hansenii* can accelerate the degradation of myogenic fibronectin and promote the production of FAAs during the later stage of sausage fermentation [101]. *L. plantarum*, *L. campestris*, and other LAB can promote protein hydrolysis to produce FAAs and peptides, resulting in a significant increase in the content of the fresh-taste substances monosodium glutamate and aspartic acid.

### 4.2. Lipolysis Metabolic Pathways

The first step in the transformation of lipids into flavor substances like FFAs is the hydrolysis of glycerol esters and phospholipids by esterase. The oxidation of FFAs produces numerous different hydroperoxides, which are subsequently decomposed through many different pathways to produce a large number of volatile compounds such as alcohols, aldehydes, acids, and alkanes, thereby providing FMFPs with a characteristic aromatic flavor [102]. Through the action of microbial enzymes, FFAs are decomposed in the process of β-oxidation to produce short-chain fatty acids and β-keto acids, which are degraded into methyl ketones and secondary alcohols through microorganisms [103]. By adding *L. plantarum* and *Staphylococcus* spp. starters to lamb sausage, Hu et al. demonstrated that the FFA content was significantly higher at the later stage of ripening than that of the early stage, so it could be suggested that microorganisms such as *Lactobacillus* promoted the oxidative degradation of lipids [104]. Similarly, inoculation of *D. hansenii* in sausage promoted the lipolysis and thus increased the FFAs, esters, and branched chain aldehydes, contributing to the production of fruit flavors [105].

### 4.3. Carbohydrate Metabolic Pathways

Carbohydrates in FMFPs are utilized by microorganisms such as LAB in order to produce a variety of aroma substances, and microorganisms primarily influence the decomposition and transformation of carbohydrates via the glycolytic pathway [106]. The carbohydrates in fermented meat are decomposed into monosaccharides by hydrolases, which are decomposed into pyruvate by glycolytic and pentose phosphate pathways. Pyruvate is oxidized and decarboxylated to produce acetyl-CoA, which is reduced to lactate under anaerobic conditions. The pyruvate decarboxylase in yeast catalyzes the decarboxylation of pyruvate to produce acetaldehyde, which is further reduced to acetaldehyde by 3-phosphoglyceraldehyde, and all these substances have certain benefits for the flavor of fermented meat [107]. In addition, *L. plantarum* can metabolize 29 carbon sources, including monosaccharides, glycosides, disaccharides, and polysaccharides, and is able to utilize a wider range of carbon sources from products. For example, by analyzing the functional genomic characteristics of *L. plantarum* ST, Yang et al. found that *L. plantarum* ST possesses a better ability to produce carbohydrate-active enzymes with abundant carbohydrate utilization capacity [108]. The organic acids produced by the metabolism of LAB are responsible for the unique sour taste of fermented meat. As time goes on, the acids and alcohols produced by LAB through the decomposition of carbohydrates are formed into esters by the catalysis of the enzymes esterase and alkyl transferase [109]. *Staphylococcus* could also be used to convert carbohydrates to organic acids to produce acetaldehyde, 2,3-butanedione, and other flavor substances. Fungi can also affect the flavor of products through carbohydrate metabolism; for instance, yeast can produce a variety of alcohols through carbohydrate metabolism [18].

## 5. Conclusions

Chinese traditional FMFPs have attracted extensive research due to their natural and pure traditional craftsmanship, as well as great regional characteristics; these include Guizhou sour fish, Xinjiang air-dried meat, Sichuan bacon, and other traditional FMFPs with great local characteristics. In this paper, we reviewed the dominant bacterial genera of eight Chinese traditional FMFPs during the fermentation process and the roles of different microorganisms in regulating the texture, color, and flavor of FMFPs.

Currently, most of the research on FMFPs concentrates on the isolation and identification of functional microorganisms, as well as on dynamic changes in flavor substances and metabolites during the fermentation process. The composition and structural characteristics of microbial communities during the fermentation process have been extensively investigated. However, the utilization of functional microorganisms isolated from Chinese traditional FMFPs has a long way to go. With the maturity of the processing technology and the rapid development of the preservation technology, the quality of FMFPs has been greatly improved. The changes in people’s diet structure and health, and the desire for high quality, have become the focus of the development of foods. Compared with foreign fermented meat, Chinese traditional FMFPs are highly characterized by Chinese regional features. Therefore, the development of unique natural starters should be the major focus in the future. That is, efforts should be made to (1) screen and isolate more functional microorganisms from Chinese traditional FMFPs; (2) utilize metabolomics and proteomics technologies to investigate the flavor formation mechanism of different fermented meats; and (3) explore the potential of utilizing microorganisms to partially replace industrial food additives for improving flavor and color.

## Data Availability

Data is contained within the article.
